# 二甲双胍通过激活腺苷酸活化蛋白激酶（AMPK）的抗肿瘤机制

**DOI:** 10.3779/j.issn.1009-3419.2013.08.07

**Published:** 2013-08-20

**Authors:** 兆煜 陈, 连唐 王, 雁扬 陈

**Affiliations:** 1 510080 广州，中山大学中山医学院 Zhongshan School of Medicine, Sun Yat-sen University, Guangzhou 510080, China; 2 510080 广州，中山大学附属第一医院病理科 Department of Pathology, the First Afliated Hospital of Sun Yat-sen University, Guangzhou 510080, China

**Keywords:** 二甲双胍, 抗肿瘤机制, 腺苷酸活化蛋白激酶（AMPK）, 代谢, 细胞周期阻滞, 氧化应激, 肿瘤干细胞转化, Metformin, Antitumor mechanism, Adenosine monophosphate-activated protein kinase (AMPK), Metabolism, Cell cycle arrest, Oxidative stress, Transformation of cancer/tumor stem cells

## Abstract

二甲双胍是一种传统的口服降糖药，临床上普遍用于2型糖尿病的治疗。近年来大量流行病学研究报道二甲双胍能够降低2型糖尿病患者的肿瘤发病率，亦有研究发现二甲双胍能在代谢途径、细胞周期、氧化应激、肿瘤干细胞转化等方面通过激活腺苷酸活化蛋白激酶（adenosine monophosphate-activated protein kinase, AMPK）信号通路，从而抑制肿瘤细胞的生长、增殖以及转化。但二甲双胍通过激活AMPK的抗肿瘤机制仍存在着争议，其确切的作用机制有待进一步深入的研究，同时亟需大规模的临床试验来证实。

## Abstract (en)

Metformin, as a traditional oral hypoglycemic agent, is commonly used in the clinical treatment for type 2 diabetes. Recently, a large number of epidemiological researches have shown that metformin could reduce the tumor morbidity of type 2 diabetes, moreover, it has also been indicated that metformin could inhibit the growth, proliferation and transformation of cancer cells in metabolic pathways, cell cycle, oxidative stress and cancer/tumor stem cells transformation via AMPK pathway activation. But the antitumor e?ect of metformin via AMPK activation still exists arguments, and the defnite mechanism remains to be further investigated and confrmed by extensive clinical trials.

二甲双胍自1957年首次应用于2型糖尿病的治疗以来，一直是2型糖尿病治疗的一线药物，其单独应用或者与其它口服降糖药物/胰岛素联合应用，发挥了独特的治疗效应。二甲双胍能够缓解胰岛素抵抗，降低机体高胰岛素水平，但不引起血糖过低，其降低血糖的作用依赖胰岛素的存在但不刺激胰岛素的分泌，也不能在功能上替代胰岛素。二甲双胍对不同的组织有不同的作用，其降糖作用主要体现在减少肝脏葡萄糖的生成、减少脂肪转化为葡萄糖、增加外周葡萄糖的利用、增加葡萄糖转化成非糖物质、改变红细胞对葡萄糖的吸收以及降低外周甘油三酯等^[1]^。自2005年Evans等^[2]^首次发现二甲双胍能明显地降低2型糖尿病患者肿瘤的发病率以来，已经有大量的流行病学研究^[3]^显示二甲双胍能降低2型糖尿病患者的胰腺癌、肝癌、结肠癌、乳腺癌、尿道癌、子宫内膜癌等肿瘤的发病率。在动物实验方面，Memmott等^[4]^以及Zhu等^[5]^的研究均报道二甲双胍能够对化学致癌药物诱导的早期肿瘤起防治作用。本篇综述将对二甲双胍激活腺苷酸活化蛋白激酶(adenosine monophosphate-activated protein kinase, AMPK)发挥抗肿瘤作用的机制进行讨论。

## 1 AMPK的结构及二甲双胍激活AMPK可能的作用机制

AMPK是一种丝氨酸/苏氨酸蛋白酶，在进化上高度保守，在能量缺乏时被激活，而在能量过度时被抑制。AMPK的激活能够促进脂肪酸的氧化，提高胰岛素的敏感性，改善高胰岛素血症和高血脂症，同时能抑制炎症反应^[6-8]^。AMPK由三个亚基组成：催化亚基(α)和两个调节亚基(β和γ)。在哺乳类动物中，这些亚基分别由2个-3个亚型组成(α1，α2；β1，β2；γ1，γ2和γ3)。AMPK α亚基上第172位苏氨酸残基磷酸化后形成的p-A MPK是A MPK活化的形式。当细胞在代谢应激下，胞内A MP的水平升高或者AMP/ATP的比例上升，AMP通过与γ亚基结合，促进α亚基上第172位苏氨酸残基的磷酸化并抑制其解磷酸化，激活α亚基从而激活AMPK^[7, 8]^。

研究报道二甲双胍通过直接和间接方式激活A MPK。二甲双胍通过阻断线粒体呼吸链复合体Ⅰ的电子传递，减少ATP的合成，使胞内A M P的水平上升，A M P/ ATP比例上升，从而间接激活AMPK^[9, 10]^。Hawley等^[11]^的研究报道二甲双胍激活完整活细胞A MPK α亚单位第172位的苏氨酸残基促使AMPK磷酸化，从而直接激活AMPK，但不能激活无细胞游离状态下的AMPK，因此认为二甲双胍可能通过作用于A MPK上游的激酶激活胞内A MPK，也有可能作为一种前体药物通过修饰作用促进AMPK形成活性形式。与Hawley等报道的二甲双胍激活AMPK α亚单位促使A MPK磷酸化不同的是，Zhang等^[12]^的研究认为二甲双胍是通过与AMPK的γ亚基结合从而激活AMPK，该研究用分子对接研究方法得出A MPK γ亚基第181位精氨酸残基、第251位苏氨酸残基和第271位苏氨酸残基可以与二甲双胍形成氢键的结论，但二甲双胍结合A MPK γ亚基后是否直接诱导AMPK磷酸化从而激活AMPK仍未知。

AMPK是肿瘤抑制因子肝激酶B1(liver kinase B1, LKB1)(也称丝氨酸-苏氨酸激酶11)的下游作用因子，能够在LKB1发生突变并失去功能时抑制肿瘤细胞的生长，同时通过提高细胞能量代谢检测点的阈值对μTOPX1、π53、脂肪酸合成酶和其它调节细胞生长和代谢的分子起作用^[13]^。但Xiao等^[14]^报道在LKB1敲除的宫颈癌细胞株中，细胞对二甲双胍的敏感性降低，在LKB1表达的情况下，二甲双胍能诱导肿瘤细胞凋亡。诱导LKB1缺陷细胞LKB1表达或者LKB1异位表达的情况下，二甲双胍能够增进AMPK的激活，细胞对二甲双胍的敏感性也增加。但至今无相关报道有直接的证据证明LKB1上存在二甲双胍的作用靶点。目前，学界普遍认为A MPK是二甲双胍的靶分子，但确切的作用位点以及激活方式尚未有明确的结论。

## 2 二甲双胍通过代谢途径抑制肿瘤的机制

AMPK是细胞能量代谢的"感受器"，激活A M PK能够抑制细胞内多种高度依赖充足三磷酸腺苷(adenosine triphosphate, ATP)供应的代谢过程，包括糖异生、蛋白质和脂肪酸的合成、胆固醇生物合成，以及促进分解代谢过程如脂肪酸β氧化和糖酵解^[15]^。

根据二甲双胍抑制肿瘤细胞代谢过程是否依赖胰岛素，将抗肿瘤机制分为依赖胰岛素的间接机制和不依赖胰岛素的直接机制。二甲双胍的间接机制有赖于激活AMPK从而抑制肝脏糖异生关键基因的转录以及促进骨骼肌摄取葡萄糖，进而降低空腹血糖和胰岛素的水平。高胰岛素水平是多种肿瘤如乳腺癌、膀胱癌、结肠癌等预后不利的因素，而二甲双胍可通过降低循环血中胰岛素水平消除这些不利因素。二甲双胍的降血糖和降胰岛素效应在抗肿瘤过程中起重要作用，因为胰岛素有促进细胞有丝分裂和延长细胞生存时间的作用，而肿瘤细胞往往表达更多的胰岛素受体，提示瘤细胞对促生长作用的激素非常敏感^[10]^。

二甲双胍不依赖胰岛素的直接作用机制源于通过LK B1激活A MPK进而抑制雷帕霉素靶蛋白(mammalian target of rapamycin, mTOR)信号通路从而抑制肿瘤细胞蛋白的合成。A MPK通过磷酸化激活肿瘤抑制基因结节性硬化复合物2(tuberous sclerosis complex 2, TSC2)，从而对mTOR起负调控作用。mTOR是多种生长因子和营养物信号的调控和整合的重要分子，同时也是磷脂酰肌醇-3-羟激酶-蛋白质激酶B/Akt(phosphatidylinositol-3-kinase-protein kinase B/Akt, PI3K-PKB/Akt)信号通路的重要分子，而PI3KPKB/Akt信号通路在人类肿瘤细胞中往往是失控的，导致肿瘤细胞的快速生长和增殖。二甲双胍激活AMPK从而抑制mTOR信号通路，引起mTOR下游的分子磷酸化失活：翻译起始因子4E结合蛋白(4E-binding proteins, 4E-BPs)和核糖体S6激酶(ribosomal protein S6 kinases, S6Ks)，抑制肿瘤细胞蛋白的合成；同时也抑制了PI3K-PKB/Akt信号通路，从而抑制肿瘤细胞的生长和增殖^[10]^。

综上所述，依赖胰岛素的间接机制强调二甲双胍作用于生物机体的整体发挥抑癌作用，而不依赖胰岛素的直接机制则认为二甲双胍直接作用于肿瘤细胞，通过激活肿瘤细胞内代谢途径的相关分子，影响代谢需要的分子来抑制肿瘤细胞的生长和增殖。

然而肿瘤细胞主要通过糖酵解途径产生能量，而正常细胞主要通过三羧酸循环产生能量，因此肿瘤细胞在缺血缺氧的应激情况下比正常细胞更具有存活和增殖的优势^[16]^。另一方面肿瘤细胞利用葡萄糖作为能源物质，其对葡萄糖的摄取和糖酵解总维持在较高的水平^[17]^。因此，AMPK的激活能够抑制肿瘤细胞的代谢过程，从而抑制肿瘤细胞快速生长和增殖所需大量能量的生成，以及减少合成其生长和增殖所需的物质，这可能是抑制肿瘤细胞生长和增殖的机制之一。但这种让肿瘤"饥饿"的方法可能只能延缓肿瘤的生长，不能最终杀灭肿瘤，这种机制在抑制肿瘤中发挥的作用还需要进一步确定。

## 3 二甲双胍通过激活AMPK减少氧化应激发挥抗肿瘤作用

活性氧(reactive oxygen species, ROS)主要包括氧离子、过氧化物和自由基等含氧元素的有机物和无机物，由于种种原因导致ROS产生增多或机体清除ROS能力的下降，机体就会出现氧化应激(oxidative stress)，可导致机体组织脂质过氧化水平升高，引起DNA氧化损伤和蛋白质的表达异常，可导致基因突变，而基因突变直接参与了细胞癌变发生过程^[18]^。二甲双胍能够抑制线粒体复合体Ⅰ，使得烟酰胺腺嘌呤二核苷酸(nicotinamide adenine dinucleotide, NADH)氧化减少，从而减少电子进入电子传递链，使得烟酰胺腺嘌呤二核苷酸磷酸(nicotinamide adenine dinucleotide phosphate, NADPH)含量的上升，进而减少ROS的产生^[19]^。肿瘤细胞的细胞色素C氧化酶异常过度活化，线粒体氧化活动选择性地增强，而细胞色素C氧化酶和NADH的活性可以被电子链转移抑制剂如二甲双胍所抑制^[20]^。Algire等^[18]^发现将细胞暴露于二甲双胍环境下，二甲双胍通过降低细胞内ROS的水平，从而减少DNA的损伤，减少细胞突变率。

关于二甲双胍是否通过激活AMPK抑制ROS的产生存在比较多的争议。Lin等^[21]^报道二甲双胍减少ROS的生成不依赖A MPK的激活，而是通过抑制信号转导与转录激活因子3 (signal transducer and activator of transcription 3, Stat3)的磷酸化以及白介素6 (interleukin-6, IL-6)的表达从而减少ROS的产生；AMPK的一个激活剂5-氨基咪唑-4甲酰胺-1β核苷(5-aminoimidazole-4-carboxamide-1β riboside, AICAR)却不能产生类似的作用，敲除LKB1-AMPK的肿瘤细胞或者mTOR受抑制的肿瘤细胞中，二甲双胍对Stat3磷酸化和IL-6表达的抑制作用不受影响，说明二甲双胍抑制IL-6/Stat3信号通路减少ROS的产生不依赖于A MPK的激活。与Lin等的报道相反，Lee等^[22]^认为二甲双胍抑制ROS的产生依赖于AMPK的激活。二甲双胍通过诱导亚铁血红素加氧酶1和内生性抗氧化剂硫氧化还原蛋白来抑制ROS的产生和NADPH氧化酶4(NADPH oxidase 4, Nox4)的表达，AMPK的抑制剂compound C以及AMPK干扰RNA能够阻断二甲双胍的上述抑制作用，AMPK的另一种激活剂AICAR产生类似于二甲双胍的抑制作用。

目前关于二甲双胍能够通过调节氧化应激阻止正常细胞或者肿瘤细胞突变已经得到大多数实验的证实，存在争议的是二甲双胍是否通过激活AMPK来调节氧化应激。Lin等^[21]^和Lee等^[22]^的研究结果之所以不同，是否因为所研究的细胞对二甲双胍的敏感性存在差异，或者是他们所研究的细胞的AMPK的表达和活化存在差别，暂时还无法确定。二甲双胍也有可能激活或者抑制AMPK信号通路上的其它分子来发挥类似于激活AMPK的作用。

与上述二甲双胍激活A MPK抑制肿瘤的报道相反，Jeon等^[23]^的研究显示能量和代谢应激状况下，胞内ATP含量下降促进AMPK的激活，但AMPK的激活非但不能杀死肿瘤细胞，反而通过氧化还原反应的调节延长肿瘤细胞的生存时间。在应激条件下，产生NADPH的磷酸戊糖的代谢途径被损害，但AMPK通过抑制乙酰辅酶A羧化酶1(acetyl-CoA carboxylase 1, ACC1)和ACC2，减少脂肪酸合成过程中的NADPH的消耗和增加脂肪酸的氧化以增加NADPH的生成，从而维持胞内NADPH的平衡。而NADPH的平衡在能量应激的情况下能降低过氧化氢的含量从而够使肿瘤细胞存活下来以及诱导肿瘤细胞的代谢适应。

上述两种观点存在分歧，可能是这些学者各自的研究条件有所差异造成的。肿瘤细胞在生长过程中对葡萄糖的摄取以及糖酵解总能维持在比较高的水平^[17]^，而Jeon等^[23]^的研究是基于实体瘤在生长过程中需要克服代谢应激，是在葡萄糖剥夺的条件下进行的研究，因而这可能是他们得出不一样的结论的原因。而其它的研究不涉及到葡萄糖剥夺，均在正常培养状态下进行研究，比较符合肿瘤在机体内的环境。另外Jeon等^[23]^的研究未涉及动物实验，其是否能在实验动物身上得到相同的结果仍未知，按照他们的研究结果可以推知缺血缺氧激活AMPK，实体瘤生长速度将比原来的快，但Memmott等^[4]^以及Zhu等^[5]^分别研究二甲双胍对化学诱癌物质诱导的肺癌和乳腺癌的抑制作用，他们的研究结果均是二甲双胍抑制了早期肿瘤的生长，但所诱导的实体肿瘤未达到肿瘤内部细胞缺血缺氧的状态，因而他们的研究与Jeon等^[23]^的研究可能没有可比性。因此二甲双胍激活AMPK与肿瘤发生发展的关系仍需更全面的研究来证实。

## 4 二甲双胍激活AMPK通过阻滞肿瘤细胞的细胞周期发挥抗肿瘤作用

二甲双胍不经由p21和p27阻滞肿瘤细胞的细胞周期并诱导其凋亡，而是通过下调细胞周期蛋白D1(cyclin D1)、细胞周期蛋白依赖性激酶4(cyclin-dependent kinase 4, CDK4)和细胞周期蛋白依赖性激酶6(cyclin-dependent kinase 6, CDK6)蛋白的表达，以及降低视网膜母细胞瘤蛋白(retinoblastoma protein, Rb)磷酸化水平，导致肿瘤细胞阻滞于G_0_期/G_1_期。Cyclin D1和pRb是细胞周期的两个主要的调节蛋白，cyclin D1通过与CDK4/CDK6结合，激活CDK4/CDK6复合体，从而使pRb磷酸化，进而使转录因子E2F释放出来，激活G1期/S期转换所需的基因。二甲双胍通过下调抗凋亡蛋白Bcl-2和Bcl-xl的表达以及上调促凋亡蛋白Bax的表达诱导肿瘤细胞的凋亡。另外，二甲双胍诱导肿瘤细胞的细胞周期阻滞于G_0_期/G_1_期，伴随着AMPK的激活以及mTOR和S6Ks的抑制，但不清楚AMPK的激活在二甲双胍阻滞肿瘤细胞周期以及诱导凋亡中发挥何种作用^[24]^。与上述报道有所不同的是，Zhuang等^[25]^报道二甲双胍诱导的细胞周期阻滞需要大量的CDK抑制因子结合CDK2和激活AMPK以及下调cyclin D1的表达量，AMPK激活后导致cyclin D1的mRNA和蛋白表达下降，缺乏cyclin D1的情况下引起对CDK抑制因子p27^Kip1^和p21^Cip1^的抑制作用降低，从而使这两种CDK抑制因子释放出来，它们通过与cyclinE/CDK2结合，进而抑制肿瘤细胞从G_1_期向S期的转化，抑制细胞的增殖。而对二甲双胍不敏感的细胞中，其p27^Kip1^和p21^Cip1^的表达水平比较低，与此相对应的是，多种类型的肿瘤不表达p27^Kip1^。Zhao等^[26]^进一步阐明二甲双胍激活AMPK信号通路阻滞肿瘤细胞的细胞周期发挥的作用：二甲双胍诱导AMPK磷酸化，从而抑制下游的mTOR，进而引起mTOR下游的靶分子40S核糖体S6激酶1 (ribosomal S6 kinase 1, S6K1)、真核细胞转录起始因子4E结合蛋白1(eukaryotic translation initiation factor 4E binding protein 1, eIF4E-BP1)、S6解磷酸化，发现cyclin D1的表达下降，同时处于G_0_期/G_1_期肿瘤细胞的比例升高，因此提出二甲双胍通过激活AMPK从而抑制mTOR，导致mTOR下游的靶分子解磷酸化，进而下调cyclin D1，将肿瘤细胞阻滞于G_0_期/G_1_期。然而Zhao等报道的证据并不充分，Chen等^[27]^用AMPK的抑制剂compound C与二甲双胍共处理肿瘤细胞或者用siRNA敲除肿瘤细胞的AMPK可以使肿瘤细胞从G_0_期/G_1_期的阻滞状态重新进入细胞周期，说明二甲双胍通过阻滞肿瘤细胞的细胞周期从而抑制肿瘤细胞的增殖依赖AMPK的激活。

也有关于二甲双胍阻滞肿瘤细胞周期不依赖A MPK激活的报道，Ben等^[28]^报道二甲双胍并不诱导细胞的凋亡，而是阻滞细胞周期使细胞停留在G_0_期/G_1_期，这种阻滞作用伴随着细胞周期蛋白cyclin D1表达量和pRb磷酸化水平的大幅度降低以及p27^kip^蛋白表达量的上升，用siRNA阻断AMPK信号通路，不能逆转二甲双胍的抗肿瘤细胞增殖的作用。因此二甲双胍阻滞肿瘤细胞周期依赖AMPK的激活和不依赖AMPK的激活这两种机制可能同时存在，但尚未有关于这两种机制共存的报道。

此外，关于二甲双胍阻滞肿瘤细胞的细胞周期的研究中，二甲双胍是否特异性的阻滞肿瘤细胞仍未知。另外Luo等^[24]^还报道肿瘤移植的动物模型中，二甲双胍干预组的肿瘤重量比对照组轻69.3%，但二甲双胍不能抑制肿瘤的继续增大，因此二甲双胍通过阻滞肿瘤细胞周期的抗肿瘤机制仍需深入研究。

## 5 二甲双胍抑制肿瘤干细胞的转化从而选择性杀死肿瘤干细胞

二甲双胍能抑制肿瘤干细胞(cancer stem cells, CSCs)的转化。CSCs转化为成熟肿瘤细胞过程中迅速出现炎症反应，CSCs的炎症因子基因的表达升高，如Let-7 miR NA家族成员IL-1α、IL-1β、IL-6和血管内皮生长因子(vascular endothelial growth factor, VEGF)基因表达升高，Stat3的磷酸化程度也增高。CSCs炎症基因表达升高与NF-κB/IκB复合体的解离有关^[29]^。NF-κB与IκB解离后进入细胞核，激活炎症相关基因，使TNF-α、IL-α、IL-1β、IL-6、IL-10等炎症基因表达增多^[30]^。多种炎症因子如IL-1α和TNF-α能激活核因子κB(nuclear factor κB, NF-κB)，氧化应激产生的ROS作为第二信使通过氧化NF-κB的半胱氨酸SH基团或者使NF-κB泛素化激活NF-κB^[31, 32]^。因此NF-κB的激活与炎症因子的表达存在正反馈的作用。NF-κB在机体的免疫和炎症反应中发挥着关键的作用，而二甲双胍能下调CSCs的炎症因子基因的表达^[29]^。二甲双胍能抑制NF-κB的活性以及抑制NF-κB下游分子IκB的磷酸化，从而抑制了NF-κB/IκB复合体的解离，CSCs的转化受到抑制。同时NF-κB能在诱导CSCs转化的15 min内被检测到，表明二甲双胍抑制CSCs的转化发生于炎症反应的触发阶段，当CSCs的炎症反应触发后再增加二甲双胍这个处理因素，发现二甲双胍不能阻断CSCs的转化，进一步说明了二甲双胍抑制CSCs的转化发生于炎症反应的早期^[29]^，反过来也说明二甲双胍抑制CSCs转化过程中的炎症反应与已表达炎症的效应分子如TNF-α、IL-α、IL-1β、IL-6、IL-10无关，可能只是通过抑制NF-κB/IκB复合体的解离从而抑制上述炎症基因的表达，从源头上抑制CSCs转化过程中的炎症反应，抑制CSCs的转化。

二甲双胍能够选择性地杀伤CSCs。CSCs与非肿瘤干细胞相比，其NF-κB的激活程度更高，而Let-7、IL-6及其它炎症因子的表达相对较低^[33]^。二甲双胍通过抑制CSCs的炎症反应选择性地杀死CSCs而对非肿瘤干细胞无此作用^[31]^。

二甲双胍与传统杀肿瘤药物联合用药明显抑制肿瘤的生长，这可能跟二甲双胍选择性杀伤CSCs有关。CSC细胞膜上大多表达ATP-cassette(ABC)家族膜转运蛋白，这类蛋白能将细胞毒素和抗肿瘤药物泵出细胞外，对多种化疗药物产生耐药性；同时CSCs大多处于细胞周期的G_0_期，通过休眠状态来逃避化疗药物的攻击；CSCs DNA修复机制的强激活从而引起治疗的失败^[34]^。二甲双胍与传统杀肿瘤药物(阿霉素、顺铂)联合用药使移植到小鼠体内的肿瘤的体积不明显增大，而单用传统杀肿瘤药物或者单用二甲双胍则肿瘤体积继续增大^[35]^，这种现象可能与二甲双胍抑制CSCs的转化有关。传统杀肿瘤药物杀灭肿瘤细胞，而CSCs继续转化为成熟肿瘤细胞，表现为肿瘤的体积仍增大，而联合二甲双胍后，二甲双胍抑制CSCs的转化，这可能是肿瘤体积不明显增大的原因。

上述这些研究都没涉及到二甲双胍是否通过激活CSCs的AMPK来抑制CSCs的转化，但二甲双胍抑制CSCs与NF-κB有关，Ji等^[36]^报道在巨噬细胞中，A MPK的激活能够抑制NF-κB的活化，因而提示二甲双胍可能通过激活CSCs中的AMPK来抑制NF-κB的活化，降低CSCs转化过程中的炎症因子水平，从而阻止CSCs的转化。二甲双胍抑制CSCs的转化过程是否激活了AMPK还有待证实。

## 6 结语

二甲双胍通过激活A MPK信号通路发挥抗肿瘤的作用得到大量研究的证实，可能存在通过代谢途径抑制肿瘤、减少氧化应激发挥抗肿瘤、阻滞肿瘤细胞的细胞周期、抑制肿瘤干细胞的转化从而选择性杀死肿瘤干细胞等几种机制，但具体机制尚未完全阐明。综上所述，二甲双胍的抗肿瘤作用可能是上述几种机制的共同作用，这几种机制互相交联，形成一个网络，共同作用于肿瘤细胞。另外流行病学所研究的是二甲双胍降低2型糖尿病患者的肿瘤发病率，其是否降低非2型糖尿病的其他患者或者健康人的肿瘤发病率仍未知，暂缺相关的前瞻性的研究结果，此种研究也存在一个难题：不患2型糖尿病的其它患者或者健康人是否愿意在未知肿瘤发生风险和非高血糖以及胰岛素抵抗的前提下服用这种降糖药物。目前二甲双胍尚未进入临床试验阶段，能否真正在人体身上发挥抗肿瘤作用亟需大规模的临床研究来验证。随着对二甲双胍抗肿瘤的认识的逐步加深，这些难题将会被陆续攻破，继而为肿瘤患者带来福音。
